# The anti-obesity effects of *Lactobacillus casei* strain Shirota versus Orlistat on high fat diet-induced obese rats

**DOI:** 10.3402/fnr.v59.29273

**Published:** 2015-12-22

**Authors:** Golgis Karimi, Mohd Redzwan Sabran, Rosita Jamaluddin, Kolsoom Parvaneh, Norhafizah Mohtarrudin, Zuraini Ahmad, Huzwah Khazaai, Alireza Khodavandi

**Affiliations:** 1 Department of Nutrition and Dietetics, Faculty of Medicine and Health Sciences, Universiti Putra Malaysia, Serdang, Malaysia; 2 Department of Pathology, Faculty of Medicine and Health Sciences, Universiti Putra Malaysia, Serdang, Malaysia; 3 Department of Biomedical Sciences, Faculty of Medicine and Health Sciences, Universiti Putra Malaysia, Serdang, Malaysia; 4 Department of Paramedical Sciences, Gachsaran Branch, Islamic Azad University, Gachsaran, Iran

**Keywords:** obesity, high-fat diet, *Lactobacillus casei* strain Shirota, orlistat

## Abstract

**Background:**

Obesity and overweight are major public health problems. Various factors, such as daily nutritional habits, physical inactivity, and genetic, are related to the prevalence of obesity. Recently, it was revealed that the gut microflora may also play an important role in weight management. Thus, this study aimed to determine the anti-obesity effects of *Lactobacillus casei* strain Shirota (LcS) compared with those of orlistat in an animal model fed a high-fat diet (HFD).

**Design:**

Thirty-two male Sprague-Dawley rats were assigned to four groups fed various diets as follows: a standard diet group, HFD group, HFD supplemented with LcS (10_8_10_9_ colony-forming units (HFD-LcS) group, and HFD group treated with Orlistat (10 mg/kg body weight)). After 15 weeks, the weights of organs, body weight, body fat mass and serological biomarkers were measured. In addition, histological analysis of the liver and adipose tissue was performed.

**Results:**

Body weight, body mass index, fat mass, leptin and glucose levels were lower, and high-density lipoprotein and adiponectin levels were higher in the HFD-LcS and HFD-orlistat groups than in the HFD group. In addition a significant difference in body fat mass was observed between HFD-LcS group with HFD-orlistat group (19.19±5.76 g vs. 30.19±7.98 g). Although the interleukin-6 level was significantly decreased in the HFD-LcS and HFD-orlistat groups compared with the HFD group, no significant change was observed in other inflammatory biomarkers.

**Conclusion:**

The results of the present study show that LcS supplementation improves body weight management and the levels of some related biomarkers. In addition, LcS supplementation showed a better result in fat mass and alanine aminotransferase reduction than Orlistat. Further studies are needed to elucidate the anti-obesity effects of LcS, with a longer period of supplementation.

Obesity and overweight are major public health problems and recently became a pandemic (1). In 2014, more than 1.9 billion adults were overweight, of whom more than 600 million were obese, worldwide (2). Bibbins-Domingo et al. (3) estimated that the prevalence of obesity will increase by 7% among men and 10% among women by 2020. Obesity and overweight increase the incidence of cardiovascular disease, stroke, type 2 diabetes (4), and several types of cancer (5, 6).

Obesity is related to poor eating habits and sedentary lifestyles. People often consume dietary supplements that can affect their health, despite providing essential nutrients like vitamins and minerals for their body. In this context, products containing probiotic microorganisms are included. Probiotics are defined as live microorganisms that can confer beneficial health effects if administered in adequate amounts. Accumulating evidence reveals a potential association between specific strains of bacteria and obesity (1, 7, 8). The structure of gut microbiota is changed in obese animals (8–11) and humans (12–14). Recent data suggest that probiotic supplements affect host nutritional metabolism, which affects energy storage, adiposity, and nutrient absorption (1, 15). Several mechanisms have been proposed that link events in the colon with the regulation of energy metabolism. However, the effects of different species of bacteria on long-term weight loss and the detailed underlying mechanisms remain unknown. *Lactobacillus casei* strain Shirota (LcS) is a bacterial strain that is commercially available as a probiotic in many countries (16) and has beneficial health effects (17, 18). In addition, drugs such as Orlistat are effective for weight management (19) by decreasing the leptin level and fat mass. However, given the high prevalence of obesity, identifying an effective treatment strategy is an ongoing struggle. Supplementing the diet with probiotics may be an alternative strategy for combating obesity and related disorders (20). Therefore, we aimed to determine and compare the effects of LcS, which was isolated from a fermented milk drink, with those of Orlistat on body weight and levels of related biomarkers in high fat diet-induced obese rats. A comparative analysis was also conducted within and between groups treated with LcS or Orlistat.

## Materials and methods

### Animals and experiments

Thirty-two 6-week-old male Sprague-Dawley rats were purchased from Central Lab Animal Inc. (Malaysia). The rats were fed a standard diet (AIN-76A, Dyets Inc., Bethlehem, PA, USA) for 2 weeks to stabilize all metabolic conditions and achieve a weight of ±200 g. Food and water were supplied *ad libitum*. Each cage contained one rat. After 2 weeks, rats were randomly selected and assigned to one of four groups (eight rats per group). Rats in group 1 were fed a standard diet (SD) and those in groups 2–4 were fed a high fat diet (HFD, 40% w/w beef tallow modified AIN-76A purified rodent diet) for 12 weeks. The nutritional content of experimental diets is shown in Table 1. This study was approved by the Animal Care and Use Committee of the Faculty of Medicine and Health Sciences, Universiti Putra Malaysia.

**Table 1:** Composition of experimental diet

Ingredients	Standard diet (g/kg diet)	Energy (kcal)	High-fat diet (g/kg diet)	Energy (kcal)
Casein	200	720	200	720
Beef tallow	0	0	400	3,600
Methionine	3	12	3	12
Starch	150	540	150	540
Sucrose	500	2,000	150	600
Cellulose	50	0	50	0
Corn oil	50	450	0	0
Vitamin mixture	10	39	10	39
Mineral mixture	35	30.8	35	30.8
Choline bitartrate	2	2	2	2
Total	1,000	3791.8	1,000	5541.8

Modified AIN-76-A diet (1).

After 12 weeks of obesity induction, rats in group 1 were assigned to the SD group, and those in groups 2–4 were assigned to the HFD group, the HFD supplemented with LcS (HFD-LcS) group and the HFD group treated with 10 mg/kg body weight Orlistat (HFD-orlistat), respectively. Rats in the HFD-LcS group were orally administered LcS [10^8^–10^9^ colony-forming units (CFU)] once per day for 15 weeks. Rats in the HFD-orlistat group were gavaged with Orlistat daily, and those in the SD and HFD groups were gavaged with water daily. Body weight was measured weekly. Body mass index (BMI) was measured at baseline (week 12) and at the end of the study (week 27), and was calculated as body weight (g) divided by the square of the anal–nasal length (cm^2^).

Waist circumference (WC; cm) was measured during anesthesia at week 12 and at the end of the study (week 27) using a standard measuring tape calibrated to 0.1 cm. Rats were placed in a recumbent position, the measuring tape was placed beneath the rat, and the measurement was taken around the transverse plane.

### Body fat and organ weights

After sacrificing the rats, body fat including retroperitoneal, mesenteric, and inguinal fat were measured. The liver, spleen, kidney, heart, and pancreas were removed and weighed using standard weighing scales calibrated to 0.1 g.

### Bacterial concentration determination

The viable plate count method was used to prepare 10^8^–10^9^ CFU of LcS to gavage the rats. Bacteria were extracted from a fermented milk drink commercially available in a supermarket and identified by 16S rRNA using a ZR Fungal/Bacteria DNA MiniPrep^TM^ Kit (catalogue number: D6005; Zymo Research, Irvin, CA, USA). After extraction, the bacterial strain was cultured in de Man, Rogosa and Sharpe (MRS) broth (Sigma-Aldrich) and incubated at 37°C for 24 h. Bacteria were harvested by centrifugation at 5,000 rpm for 5 min. After centrifuge, autoclaved normal saline was added to the isolated bacteria. The bacterial suspension was used within 15 min.

### Serological analysis

Blood samples were collected at weeks 12 and 27 by heart puncture using sterilized tube during anesthesia. Serum samples were analyzed in terms of the blood glucose level, the lipid profile, including levels of low-density lipoprotein (LDL), triglyceride (TG), high-density lipoprotein (HDL), and total cholesterol (T-chol) and liver function tests, including levels of alanine aminotransferase (ALT) and aspartate aminotransferase (AST), using HITACHI COBAS C311 reagents from Roche Diagnostics (Germany). Moreover, levels of inflammatory factors, such as interleukin 6 (IL-6), tumor necrosis factor-α (TNF-α), and C-reactive protein (CRP); pro-inflammatory factors, such as leptin; and anti-inflammatory markers, such as adiponectin, were measured using enzyme-linked immunosorbent assay (ELISA) kits according to the manufacturer's instructions (RayBio Enzyme Immunoassay Kit, USA).

### Food intake measurement

Food consumption was measured daily by subtracting the final weight in grams (i.e. weight of the empty food jar and spilt food) from the initial weight (i.e. weight of the full food jar measured on the previous day). A balance was used to weigh the food and jar.

### Histological analysis

After sacrificing the rats, the liver and adipose tissues were dissected, washed thoroughly with normal saline, trimmed, processed, and embedded in paraffin. The liver and adipose tissues were sectioned at a thickness of 4–5 µm and stained with hematoxylin and eosin (H&E). The slides were examined under a light microscope by a specialist who was blinded to the study.

### Statistical analysis

Results were presented as mean±standard error (SE). Data were evaluated for statistical significance using a one-way ANOVA. A significantly different group was identified using the least significant-difference test (LSD), which was conducted with SPSS software (SPSS, Inc.). *P*<0.05 was considered statistically significant.

## Results

### Anthropometric changes, organ weights, and food intake

**Fig. 1:**
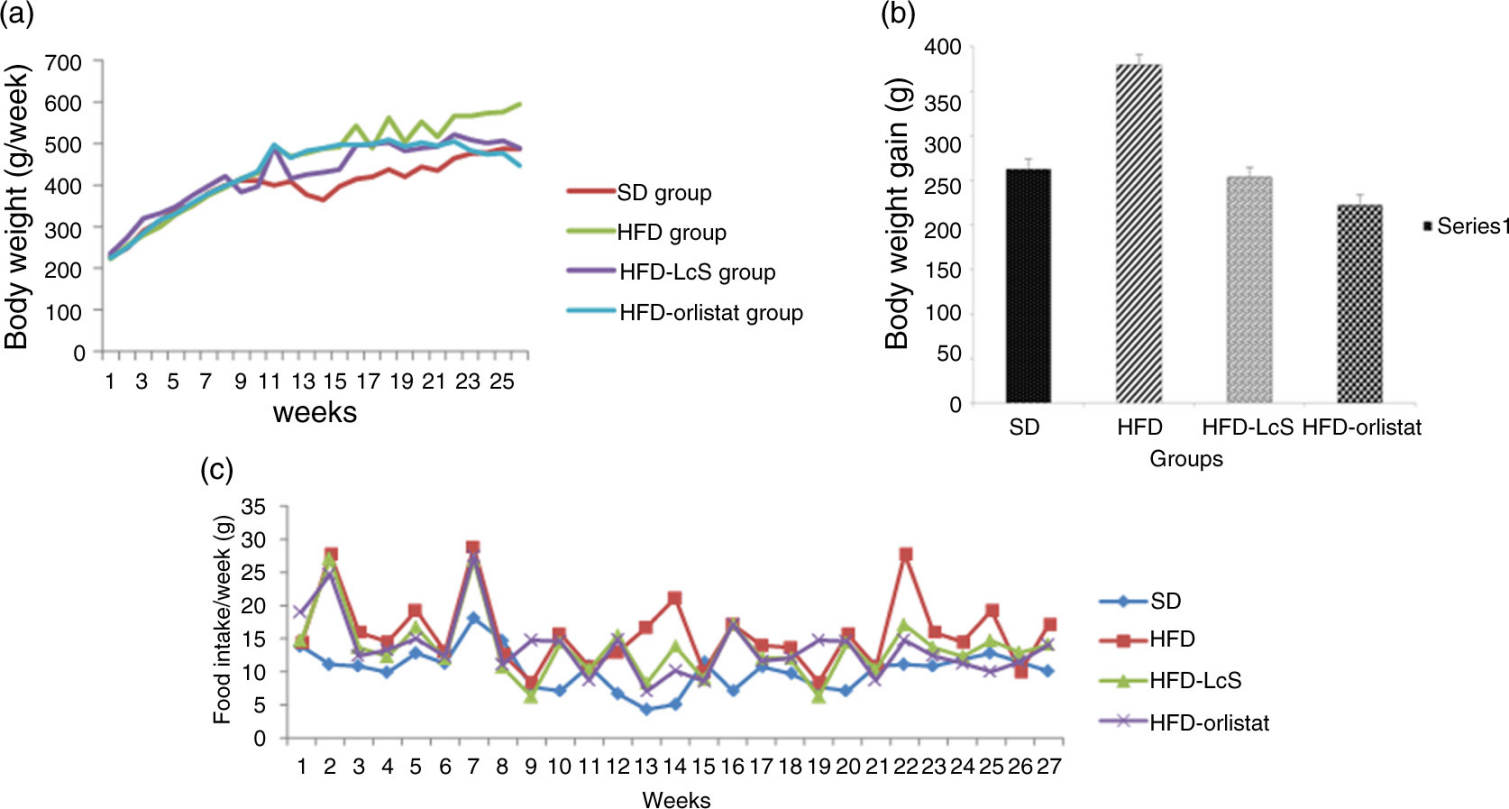
Effect of LcS on a) body weight; b) weight gain; c) food intake. SD: standard diet; HFD: high fat diet; HFD-LcS:high fat diet supplemented with LcS; HFD-orlistat: high fat diet treated with Orlistat.

An upward trend in body weight was observed in all groups every week. Within the 27-week study period, rats in the HFD group showed a significant increase in body weight (Fig. 1a). Table 2 shows the anthropometric changes (weight, height, BMI, and WC), organ weights, and fat mass of rats in the four groups. The body weight of rats in the SD and HFD groups was significantly higher at week 27 than at week 12. The HFD group exhibited the largest weight gain (379.58±11.68 g), followed by the SD group (262.3±8.45 g), the HFD-LcS group (253.21±8.10 g), and the HFD-orlistat group (221.95±5.87 g) (Fig. 1b). The BMI of rats in the HFD-LcS and HFD-orlistat groups was significantly lower at week 27 than at week 12.

**Table 2:** Effect of LcS on anthropometric, fat mass, and organs weight

	SD group	HFD group	HFD-LcS group	HFD-orlistat group
Body weight (g)				
0 week	221.56±11.43	218.25±9.08	230.22±7.69	225.25±10.8
12th week	396.22±12.29	491.63±32.32	491.63±20.22	497.13±20.55
27th week	483.86±15.72^a^	597.83±17.09^b^	488.43±7.08^a^	447.20±15.68^a^
p value within groups	0.001*	0.02*	0.89	0.11
Height (cm)	23.11±2.90	27.54±0.24	19.69±4.31	19.40±4.24
12th week	27.26±0.46	28.08±0.45	27.36±0.26	26.24±0.46
27th week	0.23	0.27	0.12	0.23
p value within groups				
Waist circumference (cm)				
12th week	14.5±0.46	17.87±0.56	16.8±0.71	17.0±0.56
27th week	18.93±0.58^b^	21.08±0.8^b^	18.38±0.57^a^	18.1±0.81^a^ ^b^
p value within groups	0.21	0.14	0.20	0.31
BMI (g/cm^2^)				
12th week	0.59±0.02	0.65±0.05	0.73±0.03	0.75±0.03
27th week	0.65±0.06^a^	0.75±0.06^b^	0.65±0.02^a^	0.65±0.06^a^
p value within groups	0.059	0.14	0.02*	0.03*
Fat mass (g)	14.81±1.62^a^	35.78±8.96^b^	19.19±5.76^a^	30.19±7.98^b^ ^c^
Liver (g)	15.7±1.08^b^	15.1±1.29^b^	11.3±0.94^a^	12.02±0.54^b^
Kidney (g)	3.21±0.421	2.87±0.67	2.53±0.57	1.59±0.61
Spleen (g)	0.72±0.107	0.58±0.13	0.76±0.23	0.49±0.19
Heart (g)	1.45±0.09	1.60±0.09	1.47±0.11	1.44±0.60
Pancreas (g)	0.509±0.203	0.561±0.188	0.560±0.11	0.536±0.172

SD: standard diet, HFD: high-fat diet, HFD-LcS: high-fat diet supplemented with LcS, HFD-orlistat: high-fat diet treated with orlistat. Data are mean±SE, SE: standard error,

* data are significantly different at week 27 compared with those in week 12 (within groups analysis, paired *t*-test).

a,b,c Data with different superscript letters are significantly different at week 27, *p*<0.05, according to the post hoc ANOVA statistical analysis (LSD), (between groups analysis).

Although the final body weight of rats in the HFD-LcS group was significantly lower than that of rats in the HFD group (488.43±7.08 g vs. 597.83±17.09 g), there was no significant difference in daily food intake between the two groups (HFD group: 15.79±1.26 g/day vs. HFD-LcS group: 13.65±2.31 g/day). Daily food intake was significantly higher in the HFD group than in the SD group (10.28±1.16 g/day, *p*<0.05) (Fig. 1c). Thus, caloric intake was higher in the HFD group, which explains the increase in body fat mass.

Moreover, rats in the HFD-LcS group had a significantly lower BMI (0.65±0.02 g/cm^2^ vs. 0.75±0.06 g/cm^2^), WC (18.38±0.57 cm vs. 21.08±0.8 cm), and fat mass (19.19±5.76 g vs. 35.78±8.96 g) than rats in the HFD group. There was no significant difference in the weight of the spleen, kidney, heart, or pancreas between the HFD-LcS group and the other groups. In contrast to body weight, BMI and WC, which did not significantly differ between the HFD-LcS and HFD-orlistat groups, body fat mass significantly differed between these two groups (19.19±5.76 g vs. 30.19±7.98 g).

### Serological analyses

Table 3 shows the blood glucose level, lipid profile (TG, HDL, LDL, and T-chol), and levels of inflammatory markers (IL-6, TNF-α, and CRP), pro-inflammatory markers (leptin), anti-inflammatory markers (adiponectin), and liver function biomarkers (AST and ALT) in each group.

**Table 3:** LcS effect on serological biomarkers (within groups and between groups analysis)

	SD Group	HFD Group	HFD-LcS Group	HFD-orlistat Group
T-cholesterol				
12th week	1.63±0.11	1.36±0.09	1.30±0.16	1.23±0.06
27th week	1.59±0.13	1.41±0.11	1.31±0.1	1.24±0.13
p	0.8	0.7	0.95	0.94
LDL-c				
12th week	0.28±0.03	0.18±0.06	0.27±0.07	0.14±0.03
27th week	0.25±0.03	0.19±0.03	0.23±0.03	0.17±0.02
p	0.55	0.81	0.58	0.48
HDL-c				
12th week	1.06±0.06	0.87±0.08	0.88±0.08	0.81±0.05
27th week	1.17±0.18	0.68±0.07^a^	1.05±0.12^b^	1.28±0.21^bc^
p	0.49	0.11	0.02*	0.013*
TG				
12th week	0.59±0.05	0.61±0.1	0.76±0.22	0.75±0.13
27th week	1.04±0.10	1.26±0.44^a^	0.83±0.15^b^	0.75±0.24^b^
p	0.001*	0.13	0.81	0.94
Leptin				
12th week	5.88±1.39	11.79±2.01	24.46±2.26	35.64±1.02
27th week	2.67±0.80^a^	13.59±2.35^b^	7.14±1.42^c^	5.02±2.90^cd^
p	0.08	0.49	0.001*	0.001*
Adiponectin				
12th week	78.022±31.48	69.71±41.60	63.59±15.49	51.71±17.18
27th week	319.4±92.31^bcd^	67.82±1.62^a^	266.98±10.9^b^	187.71±8.39^bc^
p	0.01*	0.3	0.02*	0.02*
IL-6				
12th week	66.11±2.22	74.88±3.05	93.90±7.06	87.17±6.23
27th week	105.9±18.62^a^	100.76±13.83^a^	92.72±8.93^b^	82.63±15.62^b^
p	0.013*	0.05*	0.98	0.88
TNF-α				
12th week	359.8±3.44	354.5±3.02	365.2±4.0	362.3±4.09
27th week	357.8±3.46	357.1±7.50	355.5±5.38	357.01±5.10
p	0.7	0.32	0.16	0.42
hs-CRP				
12th week	0.26±0.01	0.22±0.02	0.26±0.01	0.25±0.01
27th week	0.25±0.01	0.25±0.02	0.28±0.02	0.28±0.01
p	0.61	0.33	0.28	0.09
AST				
12th week	47.62±1.79	54.48±2.27	60.73±5.21	57.46±3.77
27th week	100.00±16.91^a^	102.90±3.20^a^	64.66±7.20^b^	65.33±10.68
p	0.003*	0.5	0.66	0.41
ALT				
12th week	20.24±1.70	24.35±3.36	33.38±4.82	26.89±4.55
27th week	31.66±6.42^a^	35.48±3.52^a^	16.47±3.51^b^	20.18±5.75
p	0.07*	0.01*	0.016*	0.3
Glucose				
12th week	6.22±0.2	6.29±0.26	8.67±0.68	9.11±1.17
27th week	9.30±1.34	10.35±1.42^a^	6.23±0.18^b^	6.15±0.35^b^
p	0.02*	0.07*	0.03*	0.01*

SD: standard diet, HFD: high-fat diet, HFD-LcS: high-fat diet supplemented with LcS, HFD-orlistat: high-fat diet treated with orlistat. Data are mean±SE. Data with different superscript letters are significantly different *p*<0.05, according to the post hoc ANOVA statistical analysis (LSD).

The serum levels of glucose, leptin, and ALT in the HFD-LcS group were significantly lower at week 27 than at week 12, with no significant changes in inflammatory biomarkers. The same trend was observed in the HFD-orlistat group. Furthermore, the adiponectin and HDL levels in the HFD-LcS and HFD-orlistat groups were significantly higher at week 27 than at week 12.

The comparison of serological biomarkers among the groups is summarized in Table 3. The levels of glucose, TG, IL-6, and leptin were significantly lower in the HFD-LcS and HFD-orlistat groups than in the HFD group at week 27, whereas the levels of adiponectin and HDL were significantly increased. The serological biomarkers did not significantly differ between the HFD-LcS and HFD-orlistat groups.

### Histological analysis

**Fig. 2:**
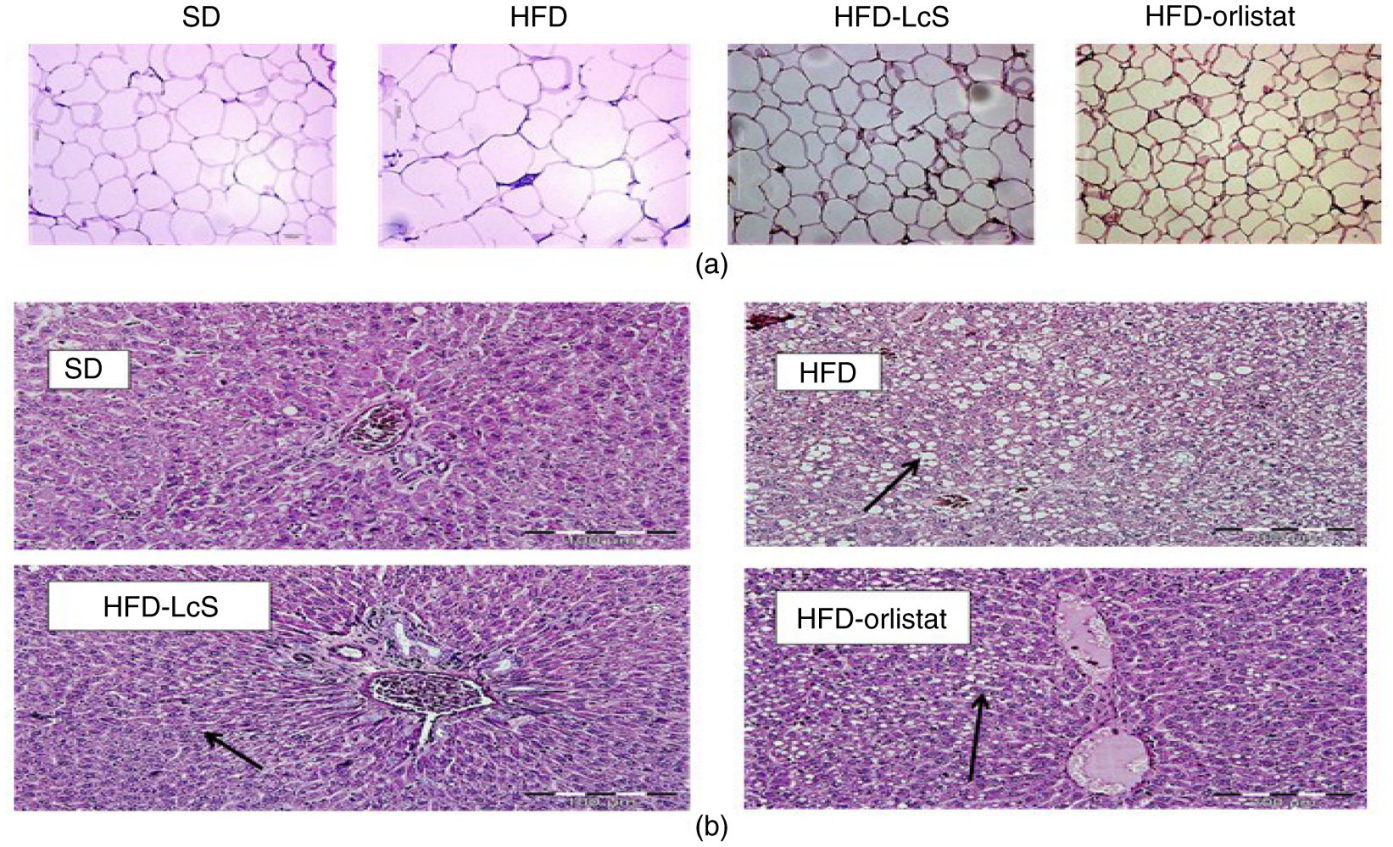
Histological analysis. a) Adipose tissue, b) liver tissue. SD: standard diet; HFD: high-fat diet; HFD-LcS: high-fat diet supplemented with LcS; HFD-orlistat: high-fat diet treated with orlistat. Size of adipocytes in SD group=59.8 µm, HFD group=374.8 µm, HFD-LcS group=156.5 µm, HFD-orlistat group=192.2 µm. Black arrows in liver tissue show the fat visuals.

H&E staining of white adipose tissue indicated that the size of adipocytes in HFD-LcS group was significantly smaller than those in the HFD group (Fig. 2a). Unlike the HFD and HFD-orlistat group, no significant macrovesicular steatosis was observed in HFD-LcS group. Furthermore, no adverse effects, such as inflammation, necrosis, and hemorrhage in hepatocytes, were observed in the liver tissue of rats supplemented with LcS (Fig. 2b).

## Discussion

### Effects of LcS on body weight, fat mass, and organs weight

Recent studies highlighted that some bacterial strains, such as *Lactobacillus* spp. and *Bifidobacterium* spp., play a role in energy metabolism and weight management in obese rats and humans (21–23). Identifying which bacterial strain should be used as a probiotic supplement is extremely important because it has been suggested that the health-promoting properties of probiotics are strain-dependent (24). Several mechanisms underlie how bacterial strains elicit their functions (21, 25, 26).

In the present study, we analyzed and compared the anti-obesity effects of the probiotic strain LcS with those of orlistat in HFD-induced obese rats. At the end of the 27-week study, HFD rats demonstrated the largest gain weight; however, rats in the HFD-LcS group had a lower body weight, fat mass, and liver weight. Body weight reduction reflects a negative status of energy expenditure, which can be due to food intake reduction or energy expenditure stimulation (27). In the present study, there was no significant difference in food intake between the supplemented groups and the control group; therefore, the body weight reduction could be due to higher energy expenditure and changes in the intestinal barrier. A HFD increased the endotoxemia through increasing the intestine permeability by reducing the expression of some genes that are encoded for tight junction proteins in the colon (28). Probiotics can increase the function of the intestinal barrier, leading to body weight loss (29). It has been also suggested that the oral administration of probiotics increases the activity of the sympathetic nervous system in white and brown adipose tissue and that the intragastric administration of probiotics increases lipolysis in white adipose tissue and thermogenesis in brown adipose tissue. Thus, probiotic consumption facilitates thermogenic and lipolytic responses via stimulating the sympathetic nervous system, which leads to weight reduction (15).

### Effects of LcS on serological biomarkers and tissue histology

The effects of LcS administration on serological biomarkers were examined. The leptin level was significantly lower in LcS-supplemented rats than in rats in the HFD group. This is consistent with the results of previous studies indicating there is an association between body and fat weight reduction and a reduced level of leptin in humans (30) and animals (1, 31). Leptin is exclusively produced by white adipose tissue (32), which acts as a global messenger to the central nervous system of systemic energy storage in order to control food intake and energy expenditure (33). Adipocyte size is an important factor for the expression of leptin and its release into blood (34). Therefore, the reduced concentration of leptin in the HFD-LcS group versus that in the HFD group may be due to a higher proportion of small adipocytes, which decreases food intake and increases energy expenditure.

Another serological biomarker that decreased in obese individuals is adiponectin, a type of adipokine that is specifically produced by adipose tissue and regulates insulin sensitivity and tissue inflammation (35). Weight reduction reportedly leads to a significant increase in the adiponectin level (36). A high level of adiponectin increases insulin sensitivity, while a low adiponectin level contributes to insulin resistance in obesity and type 2 diabetes mellitus (37). Several studies showed that probiotic supplementation may improve adiponectin secretion or expression (38–40).

The level of adiponectin was significantly increased in the HFD-LcS group after 15 weeks of supplementation, whereas the blood glucose level was significantly decreased, which is consistent with previous studies (27, 41). Moreover, the adiponectin and glucose levels were significantly different in the HFD-LcS and HFD-orlistat groups compared with the HFD group. These results are consistent with previous studies (34, 38) that demonstrated the efficacy of probiotic supplementation in terms of adiponectin and glucose secretion.

Previous studies suggest there is low-grade inflammation in obesity along with altered levels of several circulating factors, such as increases in the plasma levels of TNF-α, CRP, IL-6, and other biological markers of inflammation (42, 43). Obesity is also associated with a chronic inflammatory response, which is characterized by the activation of some pro-inflammatory signaling pathways and the abnormal production of adipokines such as leptin (44). In the present obesity model, there were no significant differences in the concentrations of inflammatory biomarkers, except for that of IL-6, which may be due to the level of obesity reached after 12 weeks. The effects of probiotics on inflammatory biomarkers are controversial. Cytokine production is assumed to be modulated by probiotics; however, this effect is strain-specific (45), possibly due to the surface antigens of bacteria (46). The comparison of inflammatory biomarkers among the groups revealed a significant reduction in the level of IL-6 in the HFD-LcS and HFD-orlistat groups, with no significant changes in the levels of TNF-α or CRP, which was consistent with the results of previous studies (46, 47).

At week 27, the HDL level was significantly higher in the HFD-LcS group than in the HFD group, with no significant changes in the other lipid profile biomarkers, which may be due to the bacterial strain. There are controversial results regarding the effect of probiotics on the lipid profile. Kang et al. (8) reported there are no significant changes in the lipid profile of rats supplemented with *Lactobacillus gasseri BNR17*. By contrast, several studies established the hypocholesterolemic effects of some bacterial strains, including *Lactobacillus acidophilus* (48) and *Bifidobacterium longum* (49).

Similar to previous studies (1, 31), the ALT level was significantly lower in the HFD-LcS group than in the HFD group. The current study also revealed that probiotic supplementation of obese rats did not affect the morphology of the liver in comparison to the control group. Serological and histological results indicated that LcS supplementation did not damage the liver.

### Anti-obesity effects of LcS vs. orlistat

Orlistat is a hydrogenated derivative of a bacterial lipase inhibitor that plays a role in body weight reduction in overweight and obese individuals (50). Previous studies indicated that receiving orlistat three times per day results in a 30% reduction in body weight in obese individuals (51). The mechanism of actions of LcS and orlistat are similar, and both reduce body weight by decreasing the leptin level and adipocyte size. In the present study, probiotic supplementation and orlistat treatment had similar anti-obesity effects. However, LcS administration showed better results in reducing fat mass and ALT level in liver.

In conclusion, we suggest that LcS, as used in this study, has beneficial anti-obesity effects but does not have anti-inflammatory or hypolipidemic effects. To the best of our knowledge, no study has compared and analyzed the anti-obesity effects of the probiotic strain LcS with those of a drug. Therefore, long-term clinical trials in humans are needed to investigate the anti-obesity effects of LcS and its related underlying mechanisms in order to explore its efficacy as an alternative treatment for obesity.

## References

[1] An HM, Lee DK, Kim JR, Cha MK, Lee SW, Lim HT (2011). Antiobesity and lipid-lowering effects of *Bifidobacterium* spp. in high fat diet-induced obese rats. Lipids Health Dis.

[2] World Health Organization (2015). Obesity and overweight. Fact sheet N°. 311, Media Centre.

[3] Bibbins-Domingo K, Chertow GM, Coxson PG, Moran A, Lightwood JM, Pletcher MJ (2010). Projected effect of dietary salt reductions on future cardiovascular disease. N Engl J Med.

[4] Golubnitschaja O, Costigliola V (2012). General report & recommendations in predictive, preventive and personalised medicine 2012: white paper of the European Association of Predictive, Preventive and Personalised Medicine. EPMA J.

[5] Gilbert CA, Slingerland JM (2013). Cytokines, obesity, and cancer: new insights on mechanisms linking obesity to cancer risk and progression. Ann Rev Med.

[6] Hursting SD, DiGiovanni J, Dannenberg AJ, Azrad M, LeRoith D, Demark-Wahnefried W (2012). Obesity, energy balance, and cancer: new opportunities for prevention. Cancer Prev Res.

[7] Million M, Angelakis E, Paul M, Armougom F, Leibovici L, Raoult D (2012). Comparative meta-analysis of the effect of *Lactobacillus* species on weight gain in humans and animals. Microb Pathog.

[8] Kang J-H, Yun S-I, Park M-H, Park J-H, Jeong S-Y, Park H-O (2013). Anti-obesity effect of *Lactobacillus gasseri* BNR17 in high-sucrose diet-induced obese mice. PLoS One.

[9] Hamad EM, Sato M, Uzu K, Yoshida T, Higashi S, Kawakami H (2009). Milk fermented by *Lactobacillus gasseri* SBT2055 influences adipocyte size via inhibition of dietary fat absorption in Zucker rats. Br J Nutr.

[10] Bäckhed F, Manchester JK, Semenkovich CF, Gordon JI (2007). Mechanisms underlying the resistance to diet-induced obesity in germ-free mice. Proc Natl Acad Sci USA.

[11] Karimi G, Jamaluddin R, Parvaneh K (2013). The effects of probiotics on body weight and biomarkers of animal. Pakistan J Nutr.

[12] Zhang Y-J, Li S, Gan R-Y, Zhou T, Xu D-P, Li H-B (2015). Impacts of gut bacteria on human health and diseases. Int J Mol Sci.

[13] Geurts L, Neyrinck AM, Delzenne NM, Knauf C, Cani PD (2013). Gut microbiota controls adipose tissue expansion, gut barrier and glucose metabolism: novel insights into molecular targets and interventions using prebiotics. Benef Microbes.

[14] Leung J, Burke B, Ford D, Garvin G, Korn C, Sulis C (2013). Possible association between obesity and *Clostridium difficile* infection. Emerg Infect Dis.

[15] Tanida M, Shen J, Maeda K, Horii Y, Yamano T, Fukushima Y (2008). High-fat diet-induced obesity is attenuated by probiotic strain *Lactobacillus paracasei* ST11 (NCC2461) in rats. Obes Res Clin Pract.

[16] Naito E, Yoshida Y, Makino K, Kounoshi Y, Kunihiro S, Takahashi R (2011). Beneficial effect of oral administration of *Lactobacillus casei* strain Shirota on insulin resistance in diet - induced obesity mice. J Appl Microbiol.

[17] Matsumoto K, Takada T, Shimizu K, Moriyama K, Kawakami K, Hirano K (2010). Effects of a probiotic fermented milk beverage containing *Lactobacillus casei* strain Shirota on defecation frequency, intestinal microbiota, and the intestinal environment of healthy individuals with soft stools. J Biosci Bioeng.

[18] Matsuzaki T, Edward RF (2003). Health properties of milk fermented with *Lactobacillus casei* strain Shirota (LcS). Handbook of fermented functional foods.

[19] Díaz EG, Folgueras TM (2011). Systematic review of the clinical efficacy of sibutramine and orlistat in weight loss, quality of life and its adverse effects in obese adolescents. Nutr Hosp.

[20] Núñez IN, Galdeano CM, de Moreno de LeBlanc A, Perdigón G (2015). *Lactobacillus casei* CRL 431 administration decreases inflammatory cytokines in a diet induced obese mouse model. Nutrition.

[21] Firouzi S, Barakatun-Nisak MY, Ismail A, Majid HA, Azmi KN (2013). Role of probiotics in modulating glucose homeostasis: evidence from animal and human studies. Int J Food Sci Nutr.

[22] Wu C-C, Weng W-L, Lai W-L, Tsai H-P, Liu W-H, Lee M-H (2015). Effect of *Lactobacillus plantarum* strain K21 on high-fat diet-fed obese mice. Evid Based Complement Alternat Med.

[23] Yoda K, Sun X, Kawase M, Kubota A, Miyazawa K, Harata G (2015). A combination of probiotics and whey proteins enhances anti-obesity effects of calcium and dairy products during nutritional energy restriction in aP2-agouti transgenic mice. Br J Nutr.

[24] Dong H, Rowland I, Yaqoob P (2012). Comparative effects of six probiotic strains on immune function in vitro. Br J Nutr.

[25] López P, Gueimonde M, Margolles A, Suárez A (2010). Distinct *Bifidobacterium* strains drive different immune responses *in vitro*. Int J Food Microbiol.

[26] Parvaneh K, Jamaluddin R, Karimi G, Erfani R (2014). Effect of probiotics supplementation on bone mineral content and bone mass density. Scientific World Journal.

[27] Stenman L, Waget A, Garret C, Klopp P, Burcelin R, Lahtinen S (2014). Potential probiotic *Bifidobacterium animalis* ssp. lactis 420 prevents weight gain and glucose intolerance in diet-induced obese mice. Benef Microbes.

[28] Cani PD, Delzenne NM (2009). Interplay between obesity and associated metabolic disorders: new insights into the gut microbiota. Curr Opin Pharmacol.

[29] Oelschlaeger TA (2010). Mechanisms of probiotic actions – a review. Int J Med Microbiol.

[30] Considine RV, Sinha MK, Heiman ML, Kriauciunas A, Stephens TW, Nyce MR (1996). Serum immunoreactive-leptin concentrations in normal-weight and obese humans. N Engl J Med.

[31] Lee H-Y, Park J-H, Seok S-H, Baek M-W, Kim D-J, Lee K-E (2006). Human originated bacteria, *Lactobacillus rhamnosus* PL60, produce conjugated linoleic acid and show anti-obesity effects in diet-induced obese mice. Biochim Biophys Acta.

[32] Ahima RS, Flier JS (2000). Leptin. Ann Rev Physiol.

[33] Zhang Y, Proenca R, Maffei M, Barone M, Leopold L, Friedman JM (1994). Positional cloning of the mouse obese gene and its human homologue. Nature.

[34] Sato M, Uzu K, Yoshida T, Hamad EM, Kawakami H, Matsuyama H (2008). Effects of milk fermented by *Lactobacillus gasseri* SBT2055 on adipocyte size in rats. Br J Nutr.

[35] Whitehead J, Richards A, Hickman I, Macdonald G, Prins J (2006). Adiponectin – a key adipokine in the metabolic syndrome. Diabetes Obes Metab.

[36] Gao X-L, Yang H-X, Zhao Y (2008). Variations of tumor necrosis factor-alpha, leptin and adiponectin in mid-trimester of gestational diabetes mellitus. Chin Med J.

[37] Feng W, Yuan X, Tong G, Wang W, Hu Y, Shen S (2013). Correlated increase of omentin-and adiponectin by exenatide, avandamet, and dietary change in diet-induced obese rats. Folia Biol (Praha).

[38] Kadooka Y, Sato M, Imaizumi K, Ogawa A, Ikuyama K, Akai Y (2010). Regulation of abdominal adiposity by probiotics (*Lactobacillus gasseri* SBT2055) in adults with obese tendencies in a randomized controlled trial. Eur J Clin Nutr.

[39] Luoto R, Laitinen K, Nermes M, Isolauri E (2012). Impact of maternal probiotic-supplemented dietary counseling during pregnancy on colostrum adiponectin concentration: a prospective, randomized, placebo-controlled study. Early Hum Dev.

[40] Nerstedt A, Nilsson EC, Ohlson K, Håkansson J, Thomas Svensson L, Löwenadler B (2007). Administration of *Lactobacillus* evokes coordinated changes in the intestinal expression profile of genes regulating energy homeostasis and immune phenotype in mice. Br J Nutr.

[41] Hulston CJ, Churnside AA, Venables MC (2015). Probiotic supplementation prevents high-fat, overfeeding-induced insulin resistance in human subjects. Br J Nutr.

[42] Das UN (2002). Is metabolic syndrome X an inflammatory condition?. Exp Biol Med.

[43] Ford ES (2003). The metabolic syndrome and C-reactive protein, fibrinogen, and leukocyte count: findings from the Third National Health and Nutrition Examination Survey. Atherosclerosis.

[44] Bastard J-P, Maachi M, Lagathu C, Kim MJ, Caron M, Vidal H (2006). Recent advances in the relationship between obesity, inflammation, and insulin resistance. Eur Cytokine Netw.

[45] Borchers AT, Selmi C, Meyers FJ, Keen CL, Gershwin ME (2009). Probiotics and immunity. J Gastroenterol.

[46] Andreasen AS, Sofie A, Larsen N, Pedersen-Skovsgaard T, Berg RM, Møller K (2010). Effects of *Lactobacillus acidophilus* NCFM on insulin sensitivity and the systemic inflammatory response in human subjects. Br J Nutr.

[47] Gøbel RJ, Larsen N, Jakobsen M, Mølgaard C, Michaelsen KF (2012). Probiotics to adolescents with obesity: effects on inflammation and metabolic syndrome. J Pediatr Gastroenterol Nutr.

[48] Park YH, Kim JG, Shin YW, Kim HS, Kim Y-J, Chun T (2008). Effects of *Lactobacillus acidophilus* 43121 and a mixture of *Lactobacillus casei* and *Bifidobacterium longum* on the serum cholesterol level and fecal sterol excretion in hypercholesterolemia-induced pigs. Biosci Biotechnol Biochem.

[49] Xiao J, Kondo S, Takahashi N, Miyaji K, Oshida K, Hiramatsu A (2003). Effects of milk products fermented by *Bifidobacterium longum* on blood lipids in rats and healthy adult male volunteers. J Dairy Sci.

[50] Dicker D, Herskovitz P, Katz M, Atar E, Bachar GN (2010). Computed tomography study of the effect of orlistat on visceral adipose tissue volume in obese subjects. Israel Med Assoc J.

[51] Blackburn GL, Waltman BA (2005). Pharmacotherapy to reduce visceral fat. Clin Cornerstone.

